# The Introduction of a BaTiO_3_ Polarized Coating as an Interface Modification Strategy for Zinc-Ion Batteries: A Theoretical Study

**DOI:** 10.3390/ijms252011172

**Published:** 2024-10-17

**Authors:** Diantao Chen, Jiawei Zhang, Qian Liu, Fan Wang, Xin Liu, Minghua Chen

**Affiliations:** Key Laboratory of Engineering Dielectric and Applications (Ministry of Education), Harbin University of Science and Technology, Harbin 150000, China; 2220300074@stu.hrbust.edu.cn (D.C.); jwzhang@hrbust.edu.cn (J.Z.);

**Keywords:** aqueous zinc-ion batteries, piezoelectric polarized BaTiO_3_, artificial solid electrolyte interphase

## Abstract

Aqueous zinc-ion batteries (AZIBs) have become a promising and cost-effective alternative to lithium-ion batteries due to their low cost, high energy, and high safety. However, dendrite growth, hydrogen evolution reactions (HERs), and corrosion significantly restrict the performance and scalability of AZIBs. We propose the introduction of a BaTiO_3_ (BTO) piezoelectric polarized coating as an interface modification strategy for ZIBs. The low surface energy of the BTO (110) crystal plane ensures its thermodynamic preference during crystal growth in experimental processes and exhibits very low reactivity toward oxidation and corrosion. Calculations of interlayer coupling mechanisms reveal a stable junction between BTO (110) and Zn (002), ensuring system stability. Furthermore, the BTO (110) coating also effectively inhibits HERs. Diffusion kinetics studies of Zn ions demonstrate that BTO effectively suppresses the dendrite growth of Zn due to its piezoelectric effect, ensuring uniform zinc deposition. Our work proposes the introduction of a piezoelectric material coating into AZIBs for interface modification, which provides an important theoretical perspective for the mechanism of inhibiting dendrite growth and side reactions in AZIBs.

## 1. Introduction

In the past three decades, research on lithium-ion batteries (LIBs) has experienced significant growth due to their high energy density, robust cycling stability, and lightweight properties [1,2,3,4]. These characteristics make LIBs increasingly de manded to meet the escalating energy requirements of modern technologies such as portable electronics, electric vehicles, and grid energy storage. However, challenges including safety concerns, high costs, and limited lithium resources restrict the widespread application of LIBs [5,6,7], prompting researchers to explore alternative energy storage devices.

Recently, aqueous zinc-ion batteries (AZIBs) have emerged as promising alternatives for economical and large-scale energy deployment due to their inherent properties [8,9,10,11]. These batteries exhibit a high specific capacity (5845 mAh/cm^−3^ and 820 mAh/g^−1^), a low redox potential (−0.76 V vs. a standard hydrogen electrode), compatibility with aqueous electrolytes, low cost, and environmental friendliness [12]. Despite these excellent attributes, AZIBs still face significant challenges such as uncontrolled zinc deposition (dendritic growth) [13], hydrogen evolution reactions (HERs) [14,15], and corrosion, which leads to poor zinc plating/stripping Coulombic efficiency (CE), low zinc electrode utilization, and a short lifespan, thereby limiting the performance of AZIBs.

High-dielectric materials, such as barium titanate, can effectively polarize the applied electric field and prevent the preferential growth of zinc dendrites [16,17,18,19,20]. Moreover, the porosity and hydrophobicity of these artificial coatings can block H_2_O at the zinc–electrolyte interface, promote the diffusion of zinc ions, and prevent the direct reaction between H_2_O molecules and the zinc surface, thereby reducing HERs and corrosion. Consequently, high-dielectric material coatings have been extensively adopted as an interface modification strategy in AZIBs. Zou et al. developed a ferroelectric BaTiO_3_ (BTO) polymer thin-film coating with a rich tunnel structure and corona polarization on the zinc anode via electroplating, achieving a horizontally aligned dense zinc morphology at ultra-high magnification [17]. Murali et al. investigated the effects of dual-peak and single-peak BTO layer coatings on zinc anodes and found that these coatings effectively reduce zinc dendrite growth in AZIBs [18]. Specifically, high-dielectric protective coatings offer unique advantages for uniform zinc deposition and suppress dendrite formation. Zong et al. developed an effective strategy combining BTO/PVDF-TrFE (BTO/PVT) coatings for dendrite-free zinc anodes [19]. The dual combination of BTO and PVT effectively suppressed dendrite growth and promoted uniform zinc deposition. Despite these advancements, current research has not yet focused on the interface modification strategy of pure high-dielectric BTO coatings on zinc anodes, and further detailed studies on the kinetics and thermodynamics of dendrite growth mechanisms are needed [20]. The above research on BTO materials only uses the large framework and special sites of BTO and does not study the special properties of BTO materials. In 2021, Zhang et al. proposed to regulate the external electric field and the built-in electric field in perovskite BTO to suppress the generation of zinc dendrites [21]. In this paper, the BTO model selected is PDF#050626. The XRD results show that the (110) surface is the most exposed surface [22]. The (110) surface is often exposed in the following ways: only Ba atoms are exposed, Ti and O atoms are exposed, or only O atoms are exposed [23].

**Figure 1:**
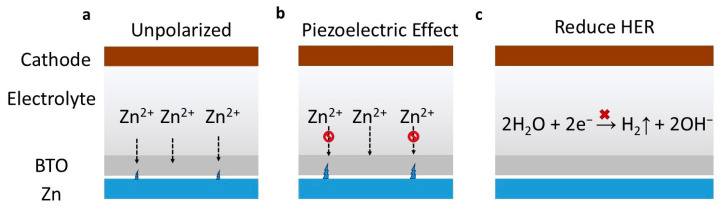
A schematic illustration of the action mechanism of an artificially introduced solid electrolyte interphase (SEI) in BTO (110). (**a**) The unpolarized phase and (**b**) the polarized phase, highlighting how dendrite formation induces interface pressure on BTO. This pressure triggers a piezoelectric effect, resulting in substantial polarization which effectively restricts Zn-ion diffusion and further suppresses dendrite growth. (**c**) A schematic representation of the HER inhibition mechanism.

In this work, we employ first-principles calculations to introduce a novel interface modification strategy for AZIBs using the BTO (110) crystal facet. The low surface energy of the BTO (110) plane ensures its thermodynamic preference during the experimental process and demonstrates enhanced resistance to interfacial reaction instability. Calculations of interlayer binding energy and charge density difference (CDD) reveal stable interfacial binding between the BTO (110) and Zn (002) heterojunction. The diffusion kinetics of Zn ions further corroborate that BTO, due to its piezoelectric effect, can effectively suppress the growth of Zn dendrites, ensuring uniform zinc deposition (Figure 1). Additionally, the BTO (110) coating exhibits a high hydrogen adsorption free energy, which effectively mitigates the hydrogen evolution reaction (HER). Our study provides a significant theoretical perspective on the use of high-dielectric materials as piezoelectric interface modifiers in aqueous zinc-ion batteries.

## 2. Results

### 2.1. Surface Energy Calculation Results

The zinc (Zn) anode is prone to the formation of unstable solid electrolyte interphase (SEI) phases which can induce ion diffusion and the deposition of Zn^2+^ at heterogeneous phase interfaces, ultimately leading to uncontrolled dendrite growth. The formation of Zn dendrites, coupled with the continuous decomposition and reformation of the SEI during repeated charge–discharge cycles, results in the loss of active Zn and a subsequent decline in Zn battery performance, thereby exacerbating interface instability. The characteristics of the SEI are closely related to the surface state of Zn metal in non-protic electrolytes. Preventing dendritic growth is crucially dependent on the construction of a robust SEI. The unstable surface has a great influence on the stability of the ASEI, so we determine the truly stable surface on the BTO (110) surface by surface energy screening.

**Figure 2:**
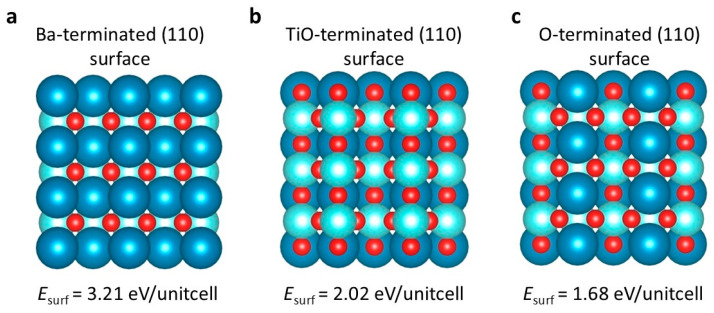
The surface energies of different terminations for the (110) surface of BTO: (**a**) the surface energy of the Ba-terminated (110) surface; (**b**) the surface energy of the TiO-terminated (110) surface; (**c**) the surface energy of the O-terminated (110) surface. The deep blue represents the barium atom, the light blue represents the titanium atom, and the red represents the oxygen atom.

A nine-layer model was used to calculate the surface energy. Computations were performed on various exposed surfaces of BTO (110) crystal facets, including (110) planes terminated with Ba, Ti-O, and O atoms. For the BTO surface, we considered two scenarios, Ba-terminated and Ti-O-terminated, due to the potential for spontaneous ionic displacement. As illustrated in Figure 2, the Ba (110) surface terminated with O atoms exhibits the lowest surface energy of 1.68 eV per unit cell. A lower surface energy ensures thermodynamic favorability for the growth of the polarized (110) plane as an SEI during experimental synthesis. Conversely, crystal facets with a higher surface energy exhibit increased activity toward oxidation and corrosion. Therefore, the (110) plane of BTO demonstrates a significant advantage as an artificial SEI due to its superior stability.

### 2.2. ASEI Binding Tightness

**Figure 3:**
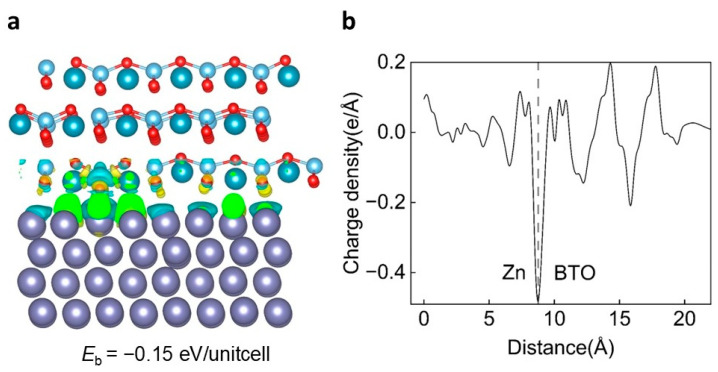
The interaction mechanism between BTO (110) and Zn (002). (**a**) A schematic of the BTO (110) and Zn (002) heterojunction, including the corresponding charge density difference (CDD) map and the interlayer binding energy between BTO (110) and Zn (002). (**b**) The plane-averaged CDD along the z direction. The observed extensive charge transfer between BTO (110) and Zn (002) layers indicates a strong Coulombic interaction. In the figure, the purple sphere is zinc atom, the dark blue is barium atom, the light blue is titanium atom, and the red is oxygen atom.

As shown in Figure 3a, we constructed BTO (110) and Zn (002) heterojunctions and studied the interaction at the interface. A binding energy of 0.15 eV/unit cell reveals that BTO (110) and Zn (002) possess a thermodynamically stable interfacial binding energy. To confirm the strong BTO (110)-Zn (002) interaction, we performed calculations to obtain the CDD between the isolated BTO (110) surface, the Zn (002) substrate, and the BTO (110)-Zn (002) complex. As shown in Figure 3a, the charge density near the topmost Zn and the downmost O is transferred to the interface, indicating a strong interaction at the BTO (110)-Zn (002) interface. We also plotted the plane-averaged CDD ∆ρ(z) along the direction perpendicular to the interface (named as z direction) (Figure 3b), which can provide a quantitative picture of the charge redistribution. It is obvious that the charge accumulation occurs at the BTO (110)-Zn (002) interface. This stability supports our hypothesis that the introduced SEI maintains a stable interface with the Zn electrode. Furthermore, the favorable interfacial characteristics of BTO (110) promote the uniform lateral dispersion of Zn, thereby suppressing the formation of dendrites in the vertical direction.

### 2.3. Inhibiting Dendrite Growth

**Figure 4:**
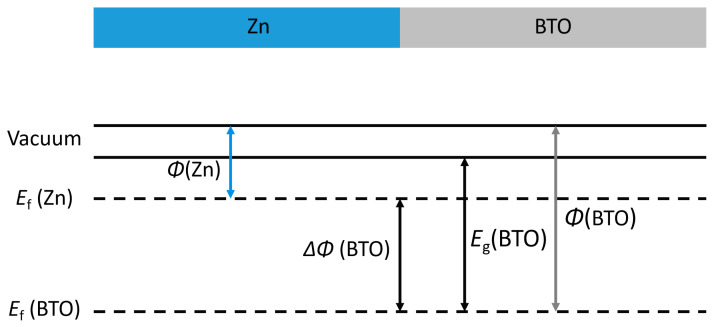
Calculation of the electron tunneling barrier (ΔE) by aligning the Fermi level (E_f_), work function (Φ), and band gap (E_g_) of the Zn anode and BTO–solid electrolyte interphase (SEI). The work function of Zn (ΦZn) is 0.76 eV, the work function of BTO (ΦBTO) is 3.49 eV, resulting in a calculated tunneling barrier of ΔE = 2.73 eV, and the band gap of BTO–bulk is 3.2 eV.

As shown in Figure 4, the excess electrons in the reaction need to overcome the electron tunneling barrier of 2.73 eV to transport to the surface and form zinc dendrites; so, the possibility of forming zinc dendrites on the surface is extremely low.

As illustrated in Figure S1, the optimal adsorption site for Zn ions in barium titanate was found within Ti-O octahedra, with an adsorption energy of −4.75 eV. This strong adsorption energy indicates a favorable interaction between Zn ions and the barium titanate structure, which is conducive to understanding the subsequent diffusion mechanisms.

Furthermore, the CI-NEB method shows the diffusion mechanism of Zn ions in piezoelectric barium titanate. Preliminary examinations of the diffusion behavior of Zn ions in unpolarized barium titanate revealed a diffusion path along the (110) crystal direction to the next Ti-O octahedron. The calculated diffusion barrier was 1.26 eV, highlighting the ease with which Zn ions can move within the crystal lattice.

**Figure 5:**
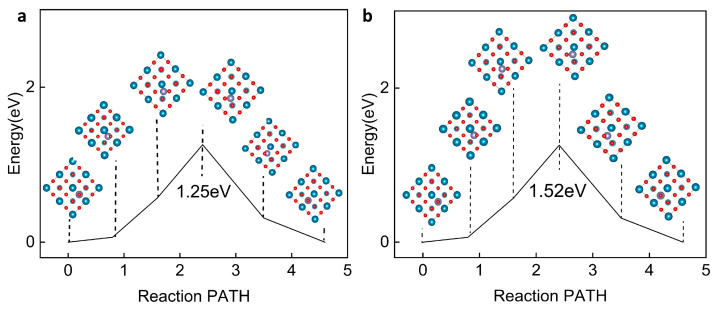
Analysis of the diffusion mechanism of Zn ions in BTO (110). (**a**) The diffusion barrier for Zn ions along the (110) direction in the non-polarized phase. (**b**) The diffusion barrier for Zn ions along the (110) direction in the polarized phase, with 5% applied strain to simulate the piezoelectric effect. It is observed that the piezoelectric effect induces significant polarization in the material, leading to a higher diffusion barrier that suppresses further growth of Zn dendrites compared to the non-polarized phase.

Subsequent analysis focused on the effect of pressure-induced macroscopic polarization on the diffusion barrier of Zn ions in barium titanate. As shown in Figure 5a, CI-NEB calculations indicate that under polarized conditions, the diffusion barrier increases to 1.52 eV, which is 270 meV higher than in the unpolarized state (Figure 5b). In the polarized phase of BTO, the migration of Zn ions along the (110) direction is significantly slower than in the unpolarized phase. This phenomenon can be attributed to several fundamental physical mechanisms. Firstly, the presence of a high polarization field induces significant lattice distortion, thereby increasing the energy barrier that Zn ions must overcome during migration. Secondly, the polarization field itself imposes constraints on the migration path of Zn ions along the (110) direction. Additionally, charge coupling in ferroelectric BTO introduces further complexity, increasing the migration energy barrier for Zn ions. These combined factors explain the observed slowdown in Zn-ion migration kinetics in the polarized phase of BTO. This finding underscores the significant impact of polarization on the diffusion properties of Zn ions in the barium titanate crystal. In summary, we conclude that after Zn ions form dendrites, the dendrites exert pressure on BTO (110), inducing a piezoelectric effect that leads to substantial polarization, thus hindering Zn ion diffusion and suppressing Zn dendrite growth.

### 2.4. Inhibiting Side Reactions

**Figure 6:**
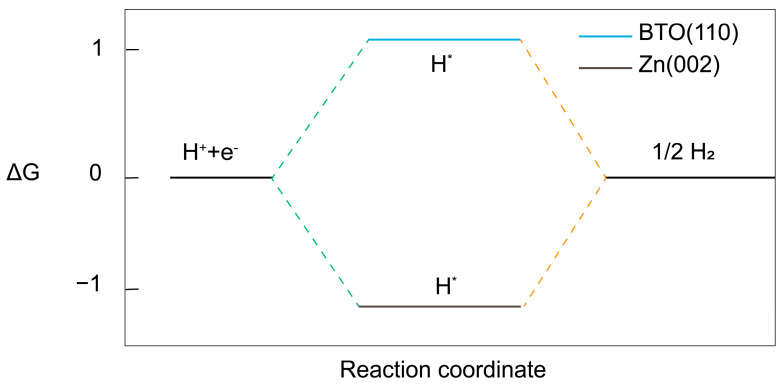
The hydrogen adsorption free energy for the HER on the BTO (110) surface and Zn (002) surface. H* represents the intermediate state of hydrogen ions adsorbed on the surface.

We investigated the impact of introducing a BTO-based SEI on the HER, revealing that the BTO (110) crystal plane exhibits exceptionally low HER activity. As illustrated in Figure 6, BTO (110) has a hydrogen adsorption free energy of 1.01 eV. For comparison, the Gibbs free energy on the Zn (002) surface is calculated as −1.34 eV. By combining the definition and comparison results of Gibbs free energy, it can be seen that the HER does not easily occur on the BTO (110) surface because the Gibbs free energy is positive, and it is difficult for the reaction to happen in the direction of hydrogen production. 

### 2.5. The Influence of Vacancy

In the actual experimental situation, due to external conditions, material processing methods, and other factors, the actual application of BTO materials in the experiment will produce vacancies. According to the analysis of the theoretical model site in Figure S2, there are 1a and 2d oxygen vacancies in the actual model. We used the CI-NEB method and differential charge density calculation to explore the effect of vacancy generation on the transport path of zinc ions and the effect of vacancy generation on the interface bonding tightness. We explored the influence of vacancies by creating vacancies on the interface of bulk materials and heterojunctions. Among them, the bulk material takes only one vacancy, and the heterojunction interface takes two vacancies, since there is only one specific vacancy on the zinc-ion transport path in the bulk material.

**Figure 7:**
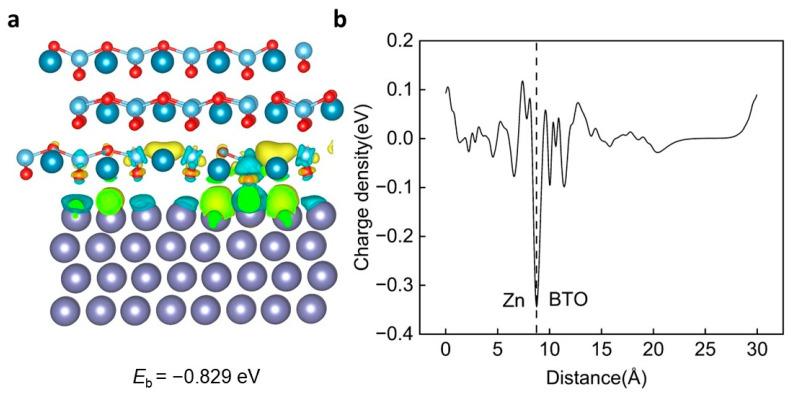
The interaction mechanism between the BTO (110) O1a vacancy and Zn (002). (**a**) The schematic diagram of the BTO (110) O1a vacancy and the Zn (002) heterojunction, including the corresponding charge density difference (CDD) diagram and the interlayer binding energy between the BTO (110) O1a vacancy and Zn (002). (**b**) The average CDD along the z direction. The observed extensive charge transfer between the BTO (110) O1a vacancy and the Zn (002) layer indicates a strong Coulomb interaction. In the figure, the purple sphere is zinc atom, the dark blue is barium atom, the light blue is titanium atom, and the red is oxygen atom.

The results of Figure 7a show that compared with the original BTO heterojunction structure, the charge exchange between the upper BTO and the lower Zn changes from uniform distribution at the interface to concentration at the vacancy, the charge distribution loses uniformity, and the degree of electron gain and loss at the vacancy is enhanced. Through the results of CDD ∆ρ(z), it can be seen that the total charge intensity is weakened, but the adsorption on the zinc surface is still strong (Figure 7b).

**Figure 8:**
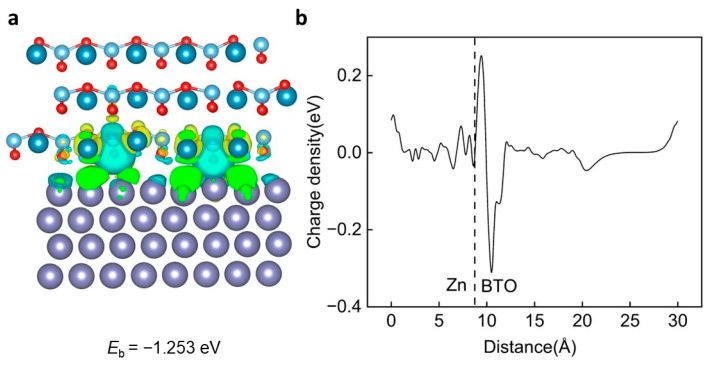
The interaction mechanism between the BTO (110) O2d vacancy and Zn (002). (**a**) The schematic diagram of the BTO (110) O2d vacancy and the Zn (002) heterojunction, including the corre-sponding charge density difference (CDD) diagram and the interlayer binding energy between the BTO (110) O2d vacancy and Zn (002). (**b**) The average CDD along the z direction. The observed extensive charge transfer between the BTO (110) O2d vacancy and the Zn (002) layer indicates a strong Coulomb interaction. In the figure, the purple sphere is zinc atom, the dark blue is barium atom, the light blue is titanium atom, and the red is oxygen atom.

However, as shown in Figure 8a, although the exposure of the 2d vacancy of O on the surface of BTO makes the charge exchange concentrated near the oxygen vacancy, it not only stays in charge exchange with specific atoms on the surface of zinc, but also has a certain charge exchange with the surrounding zinc atoms, making the exchange intensity stronger. The CDD results show that the charge loss is weaker than the vacancy-free heterojunction structure. However, there is a strong electron loss phenomenon near the BTO surface, and the adsorption effect is slightly weaker compared to the original structure, which is −1.253 eV (Figure 8b).

**Figure 9:**
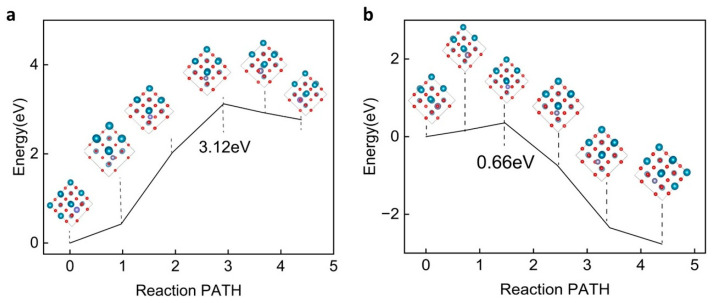
Analysis of the diffusion mechanism of Zn ions in BTO (110). (**a**) The diffusion barrier for Zn ions along the (110) O1a vacancy direction in the non-polarized phase. (**b**) The diffusion barrier for Zn ions along the (110) O2d vacancy direction in the non-polarized phase.

Figure 9a proves that the exposure of the 1a vacancy of O on the BTO surface makes the migration energy barrier decrease when the zinc ion is close to the vacancy compared with the original model, and the migration energy barrier of the movement away from the vacancy increases, up to 3.21 eV. The exposure of the 2d vacancy of O makes the migration energy barrier when the zinc ion is close to the vacancy decrease to 0.66 eV compared to the migration energy barrier of the original model. Therefore, we can conclude that the generation of the 1a vacancy is not conducive to the transport of zinc ions in BTO (Figure 9b).

## 3. Computational Methods

First-principles calculations were based on the density functional theory (DFT) and are performed by using the Vienna ab-initio Simulation Package (VASP) [24,25] with the projector augmented wave method [26]. Generalized gradient approximation with Perdew–Burke–Ernzerhof (PBE) parametrization was adopted to model exchange-correlation functionals [27]. The energy cutoff was set to 400 eV. The Brillouin zone was sampled using a Monkhorst–Pack grid with a k-mesh of 0.03. The crystal structures were fully relaxed, with energy and force convergence criteria set to 10^−5^ eV and 10^−2^ eV/Å, respectively.

### 3.1. Surface Energy

First, we analyzed the feasibility of utilizing BTO (110) as an artificial solid electrolyte interphase (ASEI). We calculated the surface energy E_surf_, which describes the stability of a surface as the energy required to cleave it from a bulk crystal, using Formula (1) [28]:

(1) $$E_{\mathrm{surf}\;}=\frac{1}{2A}(E_{s}^{unrelax}-E_{bulk})+\frac{1}{A}(E_{s}^{relax}-E_{s}^{\mathrm{un}relax})$$

Here, *A* is the area of the surface considered, *E_s_*^unrelax^ and *E_s_^relax^* are the energies of the relaxed and unrelaxed surfaces, respectively, and *E_bulk_* is the bulk energy.

### 3.2. Charge Density Difference

The stability of the ASEI itself usually easily falls off due to changes in external conditions (such as the instability of the electrode and ASEI adsorption, dendrite growth leading to ASEI rupture, etc.). Therefore, it is of great significance to explore the interface coupling dynamics between BTO (110) and Zn metal. Initially, we calculated the binding energy between the interfaces using Formula (2):

(2) $$E_{b}=E_{BTO@Zn}-E_{BTO}-E_{Zn}$$

where $E_{BTO@Zn}$, $E_{BTO}$, and $E_{Zn}$ are the energies of the whole system, the BTO (110) domain, and the Zn substrate, respectively. 

To quantitatively show the change in the charge differential density near the heterojunction, we plotted the plane-averaged CDD ∆ρ(z) along the direction perpendicular to the interface (named z direction). The plane-averaged CDD along the z direction is defined by Formula (3):

(3) $$\Delta \rho (z)=\int \rho_{BTO@Zn}(x,y,z)dxdy-\int \rho_{BTO}(x,y,z)dxdy-\int \rho_{Zn}(x,y,z)dxdy$$

where *∫ρ_BTO@Zn_(x,y,z)dxdy*, *∫ρ_BTO_(x,y,z)dxdy* and *∫ρ_Zn_(x,y,z)dxdy* are the plane-averaged charge density at the *(x,y,z)* point in the BTO (110)-Zn (002) complex, isolated BTO (110) surface, and Zn (002) substrate, respectively.

### 3.3. The Adsorption Energy and Electron Migration Energy Barrier

To gain a deeper understanding of the suppression kinetics of Zn dendrite formation by introducing BTO (110) as an artificial SEI, we conducted an extensive study on the diffusion kinetics of Zn ions in the BTO (110) plane. Initially, we explored the optimal adsorption site for Zn ions in bulk BTO through adsorption energy calculations using Formula (4):

(4) $$E_{\mathrm{ad}}=E_{BTO@Zn}-E_{BTO}-E_{Zn}$$

where *E_BTO@Zn_*, *E_BTO_*, and *E_Zn_* are the energies of the whole system, the BTO domain, and Zn atoms, respectively.

The excess electrons in the electronic conductive electrode tunnel into the electrolyte through the electronic insulator SEI film, and the electron tunneling barrier (ΔE_t_) from the negative Fermi level (ε_f_) to the bottom of the SEI film conduction band must be overcome [29,30,31]. The larger the barrier, the weaker the electron tunneling ability, which effectively inhibits the nucleation and side reactions of dendrites in the SEI film.

### 3.4. CI-NEB Method

The Climbing Image Nudged Elastic Band (CI-NEB) method was employed to investigate the diffusion mechanism of Zn ions in piezoelectric barium titanate [32]. The full name of the transition state calculation is Nudged Elastic Band (NEB), which is a method used to find saddle points and minimum energy paths on the potential energy surface between known reactants and products. The working principle of this method is to optimize many intermediate images on the reaction path. Each transition state can find as low energy as possible while maintaining the same distance from the adjacent transition states. This constrained optimization is accomplished by adding a spring force along the path between the images and a component of the projected force due to the potential effect perpendicular to the path. Unlike the NEB method, the Climbing Image Nudged Elastic Band (CI-NEB) is a small modification of the former. Since the image with the highest energy is moved to the saddle point, and it does not feel the spring force, the true strength of this image along the tangent is reversed. In this way, the image tries to maximize its energy along the path and minimize it in all other directions. When this image converges, it will be located at the exact saddle point.

## 4. Conclusions

In our study, we propose a new strategy for interfacial modification of AZIBs using piezoelectric BTO. The BTO (110) crystal plane has a low surface energy, which ensures the thermodynamic stability of the crystal growth process in the experiment, and shows very low reactivity to oxidation and corrosion. The calculation of the interfacial binding energy and CDD illustrates that the stable binding between BTO (110) and Zn (002) heterostructures originates from the strong interlayer Coulomb interaction. In addition, the analysis of the migration mechanism of Zn ions shows that BTO can effectively inhibit the growth of Zn dendrites through the piezoelectric effect mechanism and ensure uniform zinc deposition. In addition, the BTO (110) layer can effectively suppress HERs due to its greater than zero Gibbs free energy. The generation of 2d oxygen vacancies is beneficial for the transport of zinc ions in BTO materials. Our work provides a new method for interface modification of aqueous zinc-ion batteries. Introducing the polarization coating of piezoelectric materials, it provides an important theoretical perspective for the inhibition mechanism of dendrite growth in aqueous zinc-ion batteries.

## Data Availability

The raw data supporting the conclusions of this article will be made available by the authors on request.

## Supplementary Materials

The following supporting information can be downloaded at: https://www.mdpi.com/article/10.3390/ijms252011172/s1.
