# Supplementary material for: The Introduction of a BaTiO3 Polarized Coating as an Interface Modification Strategy for Zinc-Ion Batteries: A Theoretical Study
Source: Int J Mol Sci. 2024 Oct 17;25(20):11172. doi: 10.3390/ijms252011172 (PMC11508356; doi:10.3390/ijms252011172)
Supplement: Supplementary file 1 [file ijms-25-11172-s001.zip › ijms-3254207-supplementary.pdf]

Introduction of $BaTiO_3$ Polarized Coating as an Interface Modification Strategy for Zinc-Ion Batteries: A Theoretical Study

Supplementary materials

Diantao Chen, Qian Liu*, Jiawei Zhang*, Fan Wang*, Xin Liu, Minghua Chen

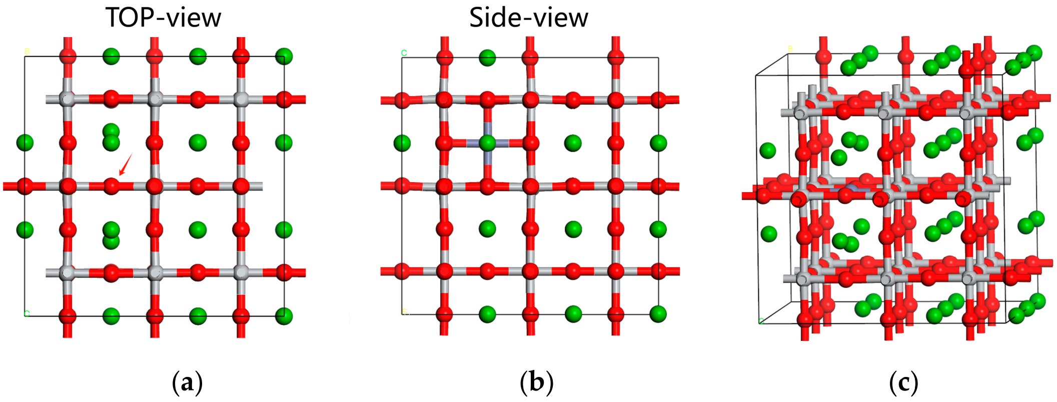

Figure S1. .Optimal adsorption sites of Zn ions in the BTO bulk: (a) top view, (b) side view, and (c) overall perspective.

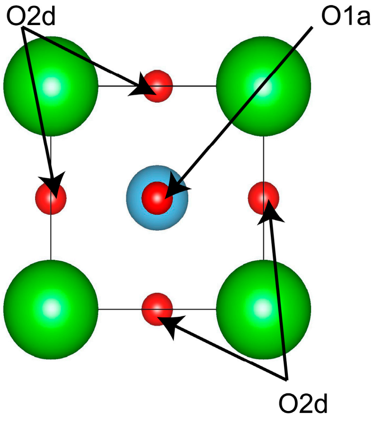

Figure S2. .Two different oxygen sites in the BTO cell, named O1a and O2d (top view)
